# Human genital dendritic cell heterogeneity confers differential rapid response to HIV-1 exposure

**DOI:** 10.3389/fimmu.2024.1472656

**Published:** 2024-10-25

**Authors:** Siddharth Parthasarathy, Laura Moreno de Lara, Francisco J. Carrillo-Salinas, Alexandra Werner, Anna Borchers, Vidya Iyer, Alison Vogell, Jared M. Fortier, Charles R. Wira, Marta Rodriguez-Garcia

**Affiliations:** ^1^ Department of Immunology, Tufts University School of Medicine, Boston, MA, United States; ^2^ Immunology Graduate Program, Graduate School of Biomedical Sciences, Tufts University School of Medicine, Boston, MA, United States; ^3^ C.S Mott Center for Human Growth and Development, Department of Obstetrics & Gynecology, Wayne State University School of Medicine, Detroit, MI, United States; ^4^ Department of Gynecology and Obstetrics, Tufts Medical Center, Boston, MA, United States; ^5^ Mass General Research Institute (MGRI), Division of Clinical Research, Massachusetts General Hospital, Boston, MA, United States; ^6^ Department of Microbiology and Immunology, Geisel School of Medicine at Dartmouth, Lebanon, NH, United States; ^7^ Department of Biochemistry, Microbiology and Immunology, Wayne State University School of Medicine, Detroit, MI, United States

**Keywords:** dendritic cell, HIV, female genital tract, single-cell RNA sequencing, mucosal immunity, antiviral response

## Abstract

Dendritic cells (DCs) play critical roles in HIV pathogenesis and require further investigation in the female genital tract, a main portal of entry for HIV infection. Here we characterized genital DC populations at the single cell level and how DC subsets respond to HIV immediately following exposure. We found that the genital CD11c^+^HLA-DR^+^ myeloid population contains three DC subsets (CD1c+ DC2s, CD14+ monocyte-derived DCs and CD14^+^CD1c^+^ DC3s) and two monocyte/macrophage populations with distinct functional and phenotypic properties during homeostasis. Following HIV exposure, the antiviral response was dominated by DCs’ rapid secretory response, activation of non-classical inflammatory pathways and host restriction factors. Further, we uncovered subset-specific differences in anti-HIV responses. CD14+ DCs were the main population activated by HIV and mediated the secretory antimicrobial response, while CD1c+ DC2s activated inflammasome pathways and IFN responses. Identification of subset-specific responses to HIV immediately after exposure could aid targeted strategies to prevent HIV infection.

## 1 Introduction

Human Immunodeficiency Virus (HIV) infection is an ongoing epidemic affecting about 38 million people worldwide. Women represent half of the people living with HIV, but in endemic regions HIV prevalence in women is higher than in men (1, 2). The main mechanism of HIV transmission is attributed to sexual intercourse (3) therefore, understanding HIV pathogenesis at the primary portal of entry, the female genital tract (FGT), remains a high priority to develop effective prevention strategies.

HIV gains access to the FGT by crossing the mucosal epithelial barrier during heterosexual transmission via seminal fluid, microtears in epithelial barriers, or transepithelial migration (4, 5). Different immune populations within the FGT supply target cells for HIV infection or act as protective innate effector cells that limit HIV acquisition (6–10). Potent antiviral innate defense followed by generation of protective local adaptive immune responses would be necessary to prevent HIV infection through repeated exposures.

Dendritic cells (DCs) are critical in shaping mucosal immunity against pathogens and maintaining tissue homeostasis (11). DCs express pattern recognition receptors (PRRs) that enable pathogen recognition and capture, specifically through C-type lectin receptors (CLRs) (12–15). Following antigen processing, DCs have the unique ability to prime naive T cell function, making DCs ideal targets for vaccination and therapeutic strategies against cancers and infections, including HIV (16). However, in HIV pathogenesis, DCs are considered a double-edged sword due to their ability to secrete anti-viral proteins and resist viral replication, but capture and transfer active viral particles to target CD4^+^ T cells (15, 17–20). Different models using *in vitro* monocyte-derived DCs, Langerhans cells and bona fide DCs have demonstrated that HIV-transfer from DCs to T cells can occur through infection-independent mechanisms via CLRs in early phases, or in an infection-dependent manner through HIV receptors and viral replication at later time points (20–24).

We previously demonstrated rapid secretion of antimicrobial peptides with anti-HIV activity by genital DCs upon HIV exposure, and subset-specific uptake of viral-like HIV particles by CD14+ DCs preferentially (25). Other studies further demonstrated that genital CD14^+^ DCs were capable of capturing and transferring HIV to CD4^+^ T cells (15, 18, 20). However, the role that different DC subsets may play in mucosal HIV acquisition remains unclear.

DCs and mononuclear phagocyte populations are highly specialized depending on the tissue of residence (26). Specifically in mucosal regions like the FGT, resident DCs display unique subset-specific functions in balancing immune protection and reproduction (27–31). Mucosal sites are populated with conventional DCs (cDCs) derived from DC precursors that seed the tissues (32). In addition, *in-vivo* recruitment and differentiation of monocytes into DCs or macrophages is mediated by acute inflammation in different disease models and at various mucosal sites (33). Characterization and delineation of DC subsets is classically dependent on surface protein expression patterns (34). However, recent advances in single-cell RNA sequencing (scRNAseq) have enabled better discrimination of human DC populations, revealing novel subsets and the inherent heterogeneity of CD14^+^ mononuclear populations (35, 36). These recent advances highlight the need to combine surface protein and RNA expression to fully characterize DC subsets (37).

Recent studies have enhanced our understanding of the DC and mononuclear phagocyte subsets that populate different human female genital mucosal surfaces relevant for HIV acquisition (15, 20, 24, 25, 31, 38, 39). Multiple of these studies identified a variety of CD14-expressing populations that remain poorly defined (40). Understanding the heterogeneity of DC and mononuclear cell populations in the FGT, along with their unique contribution to HIV pathogenesis is key for targeted interventions (41).

Here we use a combination of cellular indexing of transcriptomes and epitopes sequencing (CITE-seq) and spectral flow cytometry to define DC and mononuclear cell subsets in the FGT and determine their immediate responses to HIV exposure. Identification of phenotypic and functional properties of FGT-resident DCs and their role in HIV acquisition could inform future therapeutic and vaccination strategies against HIV.

## 2 Methods

### 2.1 Tissue processing

Tissues obtained from hysterectomies were separated by endometrium (EM), endocervix (END) and ectocervix (ECT) by pathologists and transferred to the laboratory post-surgery. Tissues were processed as described previously (10, 31, 42). Briefly, tissues were minced into 1-2 mm fragments in Roswell Park Memorial Institute (RPMI) medium (Gibco) containing enzymes from Tumor Dissociation Kit, human (Miltenyi Biotec) and 0.01% DNAse (Worthington Biochemical) and transfered into sterile gentleMACS™ C Tubes (Miltenyi Biotec). Enzymatic digestion was performed on genlteMACS™ Dissociator (Miltenyi Biotec) using “37C_h_TDK_1” program. Digested tissue was filtered through 100μm, 70μm and 30μm MACS™ SmartStrainers (Miltenyi Biotec) to generate stromal single cells suspensions.

### 2.2 Flow cytometry

Mixed single-cell suspensions from tissues were washed in Phosphate-Buffered Saline (PBS; Gibco). Subsequently, cells were incubated in the dark at room temperature with LIVE/DEAD Blue (ThermoFisher) dye for 10 minutes. Anti-CD16 (BD Biosciences) was then added and incubated for 5 minutes, followed by anti-CCR7 (BioLegend), anti-CXCR4 (BioLegend), anti-CCR5 (BioLegend) and anti-CX3CR1 (BioLegend) for another 5 minutes. Finally, cells were incubated with an antibody cocktail containing remaining antibodies (**Supplementary Table S2**) at room temperature for 15 minutes, washed with MACS buffer, fixed in 2% paraformaldehyde (PFA;Thermo Fisher), and analyzed for surface marker expression on Cytek Aurora (5 laser, 64 detector configuration; Cytek Biosciences). Expression of surface markers were quantified using OMIQ (Dotmatics).

### 2.3 HIV-1 viral stock propagation

HIV-BaL (R5) isolates obtained from the AIDS Research and Reference Reagent Program, Division of AIDS, NIAID, NIH (43) were propagated through infection of peripheral blood mononuclear cells (PBMCs) activated with phytohemagglutinin (PHA) (2.5 μg/mL; Sigma, St. Louis, MO) and IL-2 (50 U/mL; AIDS Research and Reference Reagent Program, Division of AIDS, NIAID, NIH: Human rIL-2 from Dr. Maurice Gately, Hoffmann – La Roche Inc) for 6-8 days. Stocks were harvested when p24 concentrations reached 100 ng/mL. Titration of viral stocks were performed using PHA and IL-2 activated PBMCs (44).

### 2.4 Sample preparation for multi-omics single-cell RNA sequencing

Mixed single-cell suspensions from hysterectomy samples obtained from 4 healthy female donor, consisting of 4 endometrium (EM), 2 endocervix (END) and 4 ectocervix (n =10 tissues), were enriched for immune cells by magnetic bead removal of CD3^+^ (CD3 MicroBeads, human; Miltenyi Biotec), CD19^+^ (CD19 MicroBeads, human; Miltenyi Biotec), CD235a^+^ (CD235a (Glycophorin A) MicroBeads, human; Miltenyi Biotec) red blood cells and fibroblasts (Anti-fibroblast MicroBeads, human; Miltenyi Biotec). For homeostatic conditions, cells were washed thoroughly with PBS containing 5% HS to remove any excess magnetic beads and incubated with oligo-conjugated antibodies (AbSeq; **Supplementary Table S2**) and barcoded sample tags (BD™ Hu Single Cell Sample Multiplexing Kit; BD Biosciences), to differentiate between tissue sites, for 20 minutes at room temperature in PBS containing 0.5% HS. For HIV stimulation experiments, cells were incubated with HIV-BaL (MOI = 0.5) in XVIVO-15 or media alone for 30 minutes. Cells were washed thoroughly with PBS+0.5% HS to remove unbound virus and subsequently incubated with oligo-conjugated antibodies and barcoded sample tags as mentioned above. Cells were subsequently washed twice in excess PBS+0.5% HS to remove unbound antibodies and sample tags. Cells were counted and 10,000 cells from each tissue were combined to obtain a total of 30,000 cells each for control and HIV conditions. Cells were then subsequently loaded onto separate BD Rhapsody™ Cartridge (BD Rhapsody™ Cartridge Kit; BD Biosciences) followed by RNA capture on polyA tail capture beads containing unique molecular identifiers (UMIs) (BD Rhapsody™ Enhanced Cartridge Reagent Kit V3; BD Biosciences) on BD Rhapsody™ Express Single-Cell Analysis System (BD Biosciences) followed by cDNA, whole-transcriptome amplification and single-cell indexing libraries according to manufacturer’s protocol (Protocol-Rhapsody WTA+AbSeq+ST; BD Biosciences).

### 2.5 Multi-omics RNA sequencing analysis

**Figure 1:**
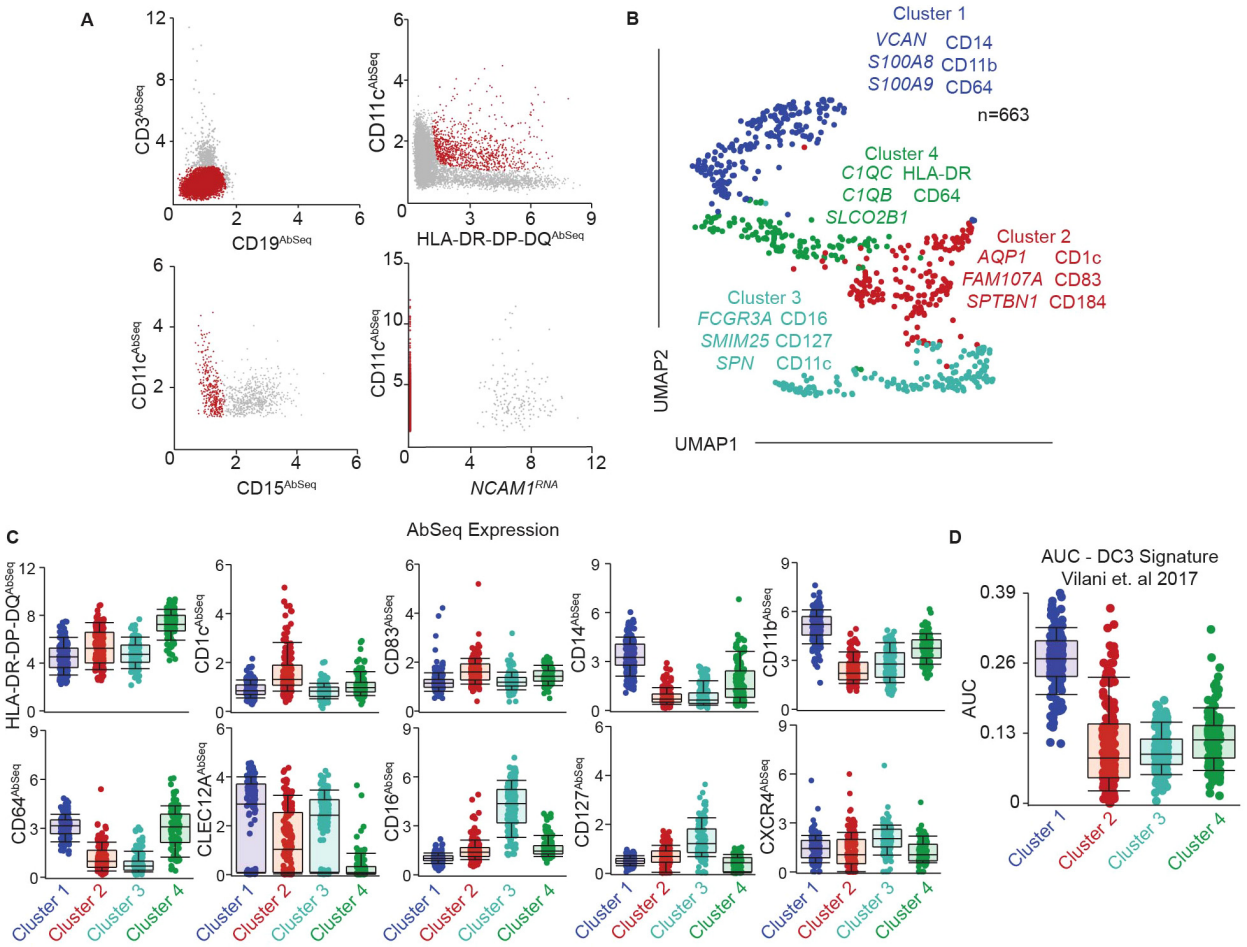
Identification of phenotypically and transcriptionally unique DC subsets in the genital tract. **(A)** Representative gating strategy to identify FGT resident CD11c^+^HLA-DR^+^ cells using a combination of surface protein expression (AbSeq) and RNA expression in CITEseq dataset. Y-axis and x-axis indicate normalized AbSeq expression levels. **(B)** Representative UMAP visualization of FGT resident CD11c^+^HLA-DR^+^ cells through unbiased clustering, depicting expression of discriminating RNA expression (left column) and surface protein expression (right column) in distinct subsets, Cluster 1 (blue), Cluster 2 (red), Cluster 3 (teal) and Cluster 4 (green). **(C)** Scatter plot comparing surface antibody expression between different FGT CD11c^+^HLA-DR^+^ subsets. **(D)** AUCell analysis of top 50 genes in DC3 cluster described by AC Villani et. al.

FASTQ files containing unaligned reads were generated on NovaSeq6000 sequencing system (Illumina). Gene counts were generated by aligning and annotating reads to the human genome (GRCh38.p12 v29). To assess capture of HIV, reads from respective experiments were also aligned to the HIV genome FASTA file. Count tables were subsequently uploaded to Partek Flow (Partek, an Illumina company) for downstream quantification and visualization of data. Cells with mitochondrial gene expression >25% number of detected features per cell less than 200 or greater than 4300 were excluded from analysis, according to recent studies published by us (31) and others (45). RNA and protein data were split and normalized respectively. To identify DCs, we first selected immune cells expressing *PTPRC* genes, which encodes CD45 protein. Next, we used protein information to exclude remaining T and B cells by selecting CD3^-^CD19^+^ cells, followed by selection of CD11c+HLA-DR-DP-DQ+ cells. Since genital NK cells and neutrophils can express CD11c and HLA-DR (46, 47), we excluded neutrophil contamination by selecting CD15- cells within this population, and NK cell contamination by gating out cells expressing *NCAM1* RNA (**Figure 1A**). PCA was performed on normalized data followed by UMAP for visualization. Additionally, we also performed weighted-nearest neighbor analysis to integrate protein and RNA data. Subsequently, we performed k-nearest neighbor analysis and graph-based analysis to identify cell clusters and used “compute biomarker” function in Partek Flow (Partek – an Illumina company) to generate biomarkers associated with the clusters (**Supplementary Data Sheet S1**). To identify differentially expressed genes (DEGs) between DC subsets, we performed non-parametric ANOVA to identify significantly upregulated and downregulated genes with significance of p ≤ 0.05 and Log_2_(FoldChange) ± 1.2. GO Biological processes (48, 49) and Reactome (50) were generated using gene lists of upregulated and downregulated genes. To identify unique expression of genes within each cluster, “compute biomarker” function in Partek Flow Analysis. Curated gene lists were used to assess expression of CD markers, cytokines, chemokines, PRRs, antimicrobial proteins and gene lists associated with GO processes for AUCell (51) (**Supplementary Data Sheet S2**) were used from GSEA analysis available in Partek Flow (Partek – an Illumina company).

### 2.6 CD14^+^ DC isolation and HIV stimulation

CD14^+^ DCs were isolated from mixed-cell suspensions using magnetic bead separation (CD14 MicroBeads, human; BD Biosciences) as per previous studies (25, 30, 31, 42). Isolated cells were incubated at 37°C, 5% CO_2_ with HIV-1-BaL (R5) isolates (MOI = 0.5) for 3 hours. Supernatants were collected and stored at -80C until multiplex assay analysis to determine secreted protein levels.

### 2.7 Luminex assay

Supernatants from unstimulated controls and HIV exposed CD14+ DCs were quantified using Millipore human cytokine multiplex kits according to manufacturer’s instructions. Signal was measured using Magpix software with five-parametric-curve fitting for data analysis. Molecules measured included IL-1β, IFNγ, IL-5, IL-13, IL1Rα, GM-CSF, G-CSF, CCL11, CCL22, CXCL1, CXCL10 and CX3CL1.

### 2.8 Statistical analysis

To identify differentially expressed genes, significance threshold was set as (p<0.05; -1.2 <FC>1.2) on non-parametric ANOVA. Statistics for flow cytometry analysis were done using Friedman’s multiple comparison, (p ≤ 0.05 *; p ≤ 0.01 **; p ≤ 0.001 ***; p ≤ 0.0001 ****). Hierarchical clustering heatmaps were performed on significant, differentially expressed genes, by generating pseudo-bulk expression of groups (eg. HIV vs control; cluster 1, cluster 2, cluster 3, cluster 4 comparison) and visualized using bubble plots, with size of the bubble referring to percentage of cells expressing the particular gene, shade and color referring to Z-score expression of the gene. For Reactome and GO analysis, terms with FDR ≤ 0.05 were chosen as significant values and bubble plots were visualized using GraphPad Prism.

### 2.9 Study subjects

Written informed consent was obtained before surgery from HIV-negative women undergoing hysterectomies at Tufts Medical Center (Boston, MA, USA). Studies were approved by Tufts University Institutional Review Board and the Committee for the Protection of Human Subjects. Surgery was performed to treat benign conditions including fibroids, prolapse, and menorraghia. Trained pathologists selected tissue samples from endometrium (EM), endocervix (END), and ectocervix (ECX), free of pathological lesions and distant from the sites of pathology. Women were HIV− and HPV− but no additional information regarding other genital infections was available.

## 3 Results

### 3.1 Identification of phenotypically and transcriptionally unique DC subsets in the genital tract

Prior studies have identified different genital DC and mononuclear phagocyte populations within the anogenital mucosa (14, 15, 20, 24, 25, 31, 39, 42, 52). However, transcriptional, phenotypical, and functional characterization of the heterogenous CD14-expressing populations in the genital tract remains poorly defined.

To address these gaps, we optimized a protocol to define DC subsets in the FGT phenotypically and transcriptionally at the single-cell level. We generated single cell suspensions from human hysterectomy samples and enriched the target immune cells as detailed in methods. We labeled the enriched cells with oligo-conjugated antibodies targeted towards surface proteins (**Supplementary Table S1**), lysed the cells to release RNA, and sequenced the RNA to simultaneously determine the whole transcriptome profile and surface protein expression at the single-cell level. To identify the genital resident DCs, we took advantage of CITE-seq’s combined surface protein information and gene expression and developed a gating strategy to select the CD11c^+^HLA-DR^+^ population (**Figure 1A**), which contains DCs. This approach eliminates the need for lengthy fluorescence activated cell-sorting (FACS) prior to sequencing, thereby limiting processing steps that can alter primary tissue resident immune cells (53).

Unbiased clustering analysis discriminated four distinct clusters of CD11c^+^HLA-DR^+^ cells within the genital mucosa (**Figure 1B**). We determined the top RNA and surface protein (Abseq) expression unique to each cluster by using “compute biomarkers” function (**Figure 1B**). Cluster 1 was characterized by RNA expression of *VCAN*, *S100A8*, *S100A9* and surface protein expression of CD14, CD11b and CD64 (**Figure 1C** **),** confirming the presence of a CD14^+^ monocyte-derived DC population in the genital mucosa as previously described by us and others (20, 25, 30, 31, 52, 54). Cluster 2 was characterized by surface protein expression of CD1c, the DC maturation marker CD83, and the pro-survival and tissue homing marker CXCR4 (55, 56), suggesting enrichment of cDC2s in this cluster (**Figure 1C**). Clusters 3 and 4 displayed phenotypes similar to monocyte populations. Cluster 3 expressed elevated CD16 and CD127, whereas cluster 4 displayed elevated expression of CD64, HLA-DR and low levels of CLEC12A protein expression (**Figure 1C**). Additionally, cluster 3 expressed RNA transcripts such as *FCGR3A*, *SMIM25* and *SPN (* **Figure 1B** *)*, consistent with non-classical monocyte (NCM) signature expression (57). Cluster 4 expressed elevated levels of complement encoding RNA transcripts *C1QC* and *C1QB*, consistent with inflammatory monocytes/macrophages (infMons) (58). These two clusters indicated the presence of two distinct monocyte populations within CD11c^+^HLA-DR^+^ cells.

Recent studies identified a novel DC population in peripheral blood named DC3 that co-expresses CD14 and CD1c and displays and intermediate phenotype and function between monocytes and classical myeloid DCs (cDC2s) (36, 59). Interestingly, we and others have previously described the presence of CD14^+^CD1c^+^ cells in the FGT (20, 25, 52). To determine whether the DC3 population was present in the FGT, we analyzed our CITE-seq data using AUCell (51) with the top 50 genes described by Villani et al. in their peripheral blood DC3 cluster (**Supplementary Data Sheet S2**) (35). We observed an enrichment of the DC3 signature in cluster 1 (**Figure 1D**), indicating the presence of DC3s within the CD14^+^ DC cluster.

Overall, our multi-omics data indicates the presence of four distinct CD11c^+^HLA-DR^+^ populations in the FGT, consisting of two DC and two monocyte populations, with unique transcriptional and phenotypic signatures. Furthermore, we observed the presence of novel DC3 gene signature in the CD14^+^ DC cluster, suggesting inherent heterogeneity of CD14^+^ DCs within the FGT.

### 3.2 Functional specialization of genital DCs under homeostatic conditions

Next, we investigated whether our phenotypical clustering was associated with distinct subset-specific functions for each subset under homeostatic conditions.

**Figure 2:**
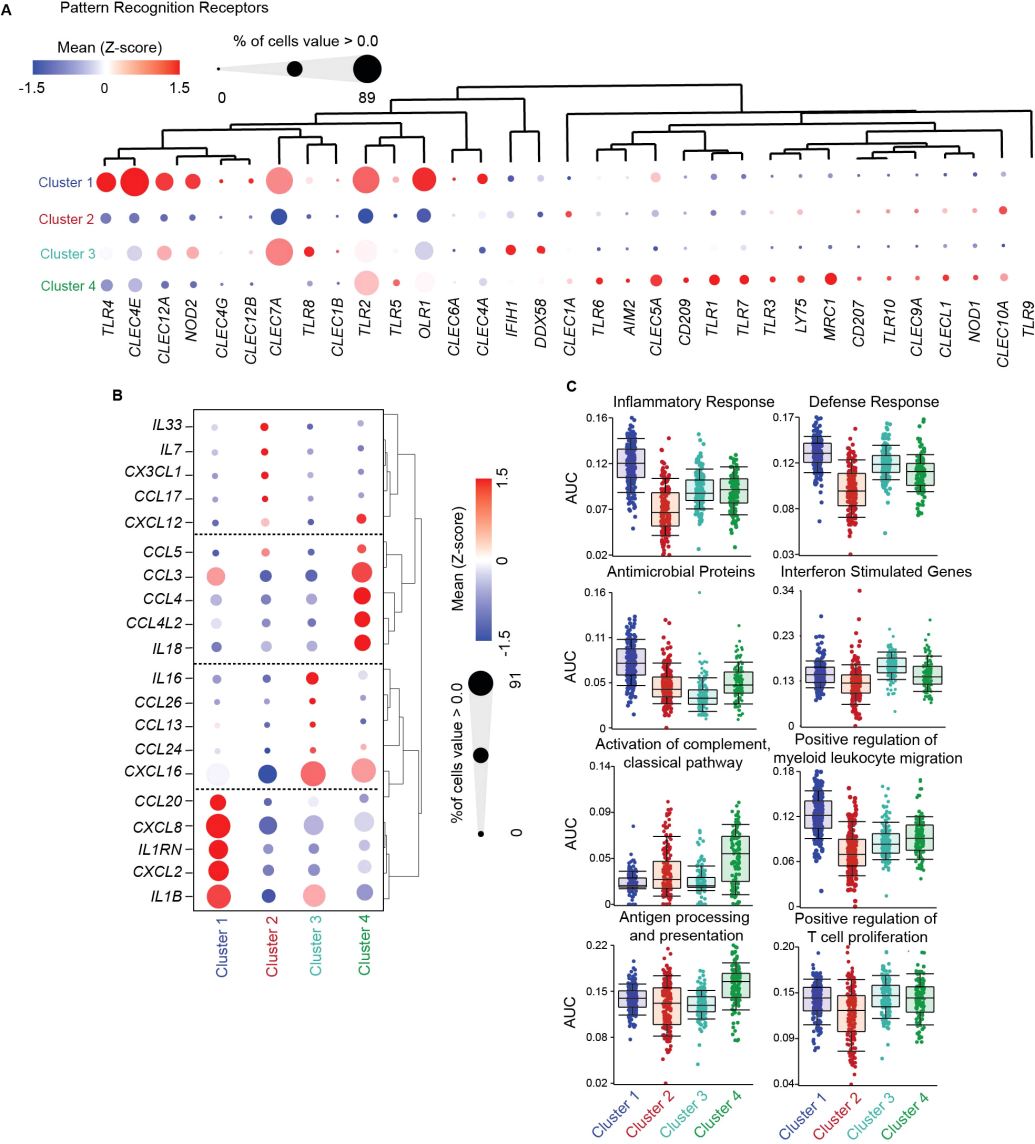
Functional specialization of genital DCs under homeostatic conditions. **(A)** Hierarchical clustering heatmap comparing gene expression of significant pattern recognition receptors (PRRs) between FGT resident CD11c^+^HLA-DR^+^ subsets. **(B)** Hierarchical clustering heatmap comparing gene expression of top 5 interleukin and chemokines in each cluster, where size of the circle indicates percentage of cells expressing associated gene > 0 **(C)** AUCell comparison of key gene ontology (GO) terms associated with dendritic cell function between DC subsets.

To determine if the distinct populations would be differently posed to detect and interact with invading pathogens, including HIV, we compared expression levels of PRRs (**Supplementary Data Sheet S2**) including toll-like receptors (TLRs), C-type lectin receptors (CLRs) and NOD-like receptors (NLRs). Using a list of known PRRs (**Supplementary Table S2**) we compared expression levels between subsets through hierarchical clustering (**Figure 2A**). We observed no TLR9 expression across all subsets, ruling out the possibility of plasmacytoid DC contribution to the gene signature of each subset. Compared to the other clusters, Cluster 1 (CD14^+^ DCs) showed relatively higher expression of TLRs and CLRs associated with detecting and binding to HIV, including *CLEC4A*, *CLEC4E* and *TLR4* (60–62). Additionally, in Clusters 1 and 3, we observed shared expression of *CLEC12A*, which has been linked to improved antigen delivery and cross presentation by DCs (63), and *CLEC7A*, previously described in CD14^+^ DCs from blood and lungs (64). Additionally, Clusters 1, 3 and 4 also shared expression of *TLR2*, which binds HIV *(* 65 *)*. Cluster 2 (cDC2s) had no unique gene expression pattern. The highest expression in Cluster 2 was observed for the CLRs *CLEC10A* and CLEC1A, involved in antigen internalization and presentation (66, 67). When compared to the other clusters, Cluster 3 (NCM) displayed high preferential expression of *IFIH1* (encoding MDA5), *DDX58* (encoding RIGI) which mediate detection of cytosolic viral RNA (68, 69), and *TLR8*, which recognizes endolysosomal ssRNA (70). Cluster 4 (infMons) expressed *MRC1* (encoding mannose receptor (MR)) and *CD209*, consistent with a monocyte/macrophage phenotype (71, 72). Cluster 4 also expressed high levels of *CLEC5A* which forms heterodimers with CD209 and MR upon antigen exposure to enhance viral internalization (73). Further, compared to the other clusters, Cluster 4 had preferential expression of *TLR1* and *TLR6*, known to dimerize with TLR2 for detection of bacterial lipopeptides; *TLR7* and *TRL3* which recognize ssRNA and dsRNA, respectively (74, 75). Finally, a small percentage of cells in Cluster 4 preferentially expressed *AIM2*, involved in recognition of cytosolic dsDNA (76). Altogether, our analysis reveals subset specific differences in genital DC recognition of different moieties of pathogens, Cluster 1 being enriched with PRRs associated with membrane HIV recognition and binding, Cluster 2 expressed PRRs for antigen uptake, Cluster 3 and Cluster 4 were enriched for PRRs that detects cytosolic and endolysosomal ssRNA, dsRNA and dsDNA.

Using the same analytical approach, we next focused on cytokine and chemokine expression under homeostatic conditions, to understand functional differences in innate secretory functions involved in maintenance of mucosal barrier integrity, immune cell recruitment and anti-microbial protection. We identified the top 5 cytokine and chemokine gene signatures unique to each cluster (**Figure 2B**; **Supplementary Data Sheet S2**). Cluster 1 (CD14^+^ DCs) was characterized by *CCL20, CXCL8* and *CXCL2* expression, genes involved in antimicrobial activity (77), chemotaxis for Th17 cells, neutrophils, monocytes and DCs (78–81). Expression of *IL1B* and its inhibitory receptor *IL1RN* were also elevated in Cluster 1, suggesting production of IL1β but prevention of autocrine/paracrine actions of IL1β on CD14^+^ DCs. Cluster 2 (containing cDC2s), specialized in expression of genes involved in T cell chemotaxis: *IL33*, an alarmin that controls tissue homeostasis and type 2 immunity (82); *CCL17*, a chemoattractant for helper T cells expressing CCR4 (such as Th2 and regulatory T cells) (83) (84); *CXCL12*, a CXCR4-ligand; *CX3CL1* (fractalkine) which play critical roles in menstruation (85); and *IL7*, suggesting an important role for cDC2s in control of genital T cell populations and favoring Th2 and T regulatory profiles. Cluster 3 (NCMs) expressed high levels of *IL16*, a cytokine that exclusively binds and signals through CD4 (86); and *CXCL16*, important for control of MAIT cells and NK cells and expressed by non-classical monocytes in blood (87–89). In addition, a smaller percentage of cells in cluster 3 expressed CCR3 ligands *CCL24*, *CCL26 and CCL13*, with antimicrobial properties and potent chemoattractants for eosinophils and basophils (90) (91). Cluster 4 (infMons) was characterized by expression of CCR5 ligands *CCL3*, *CCL4, CCL5* and CXCR4 ligands *CXCL12* and *CCL4L2*, suggesting a potential role in mediating anti-HIV activity. Cluster 4 also expressed high levels of IL18, a proinflammatory cytokine described in tissue-resident macrophages that induces IFN-γ production by T cells in an inflammatory environment, or Th2 differentiation in the absence of inflammatory cytokines (92, 93).

To further understand functional contributions to mucosal homeostasis and defense, we performed AUCell analysis (51) of genes associated with immune protection (**Figure 2C**; **Supplementary Data Sheet S2**). Cluster 1, containing CD14^+^ DCs, was enriched for genes associated with inflammatory and defense response, suggesting enhanced potential to mediate innate immune protection. Furthermore, enrichment of genes associated with positive regulation of myeloid leukocyte migration within this cluster suggests the presence of a migratory DC population. Cluster 2 (cDC2s) and Cluster 4 (infMon) were enriched for genes related to activation of the complement classical pathway, correlating with increased expression of complement associated genes. Lastly, no differences were observed for enrichment of antigen processing and presentation or positive regulation of T cell proliferation genes in genital CD11c^+^HLA-DR^+^ subsets under homeostatic conditions.

Overall, our data demonstrates distinct functional specialization of genital CD11c^+^HLA-DR^+^ subsets under homeostatic conditions.

### 3.3 CD14^+^ DCs represent a heterogenous, activated population within the genital mucosa

**Figure 3:**
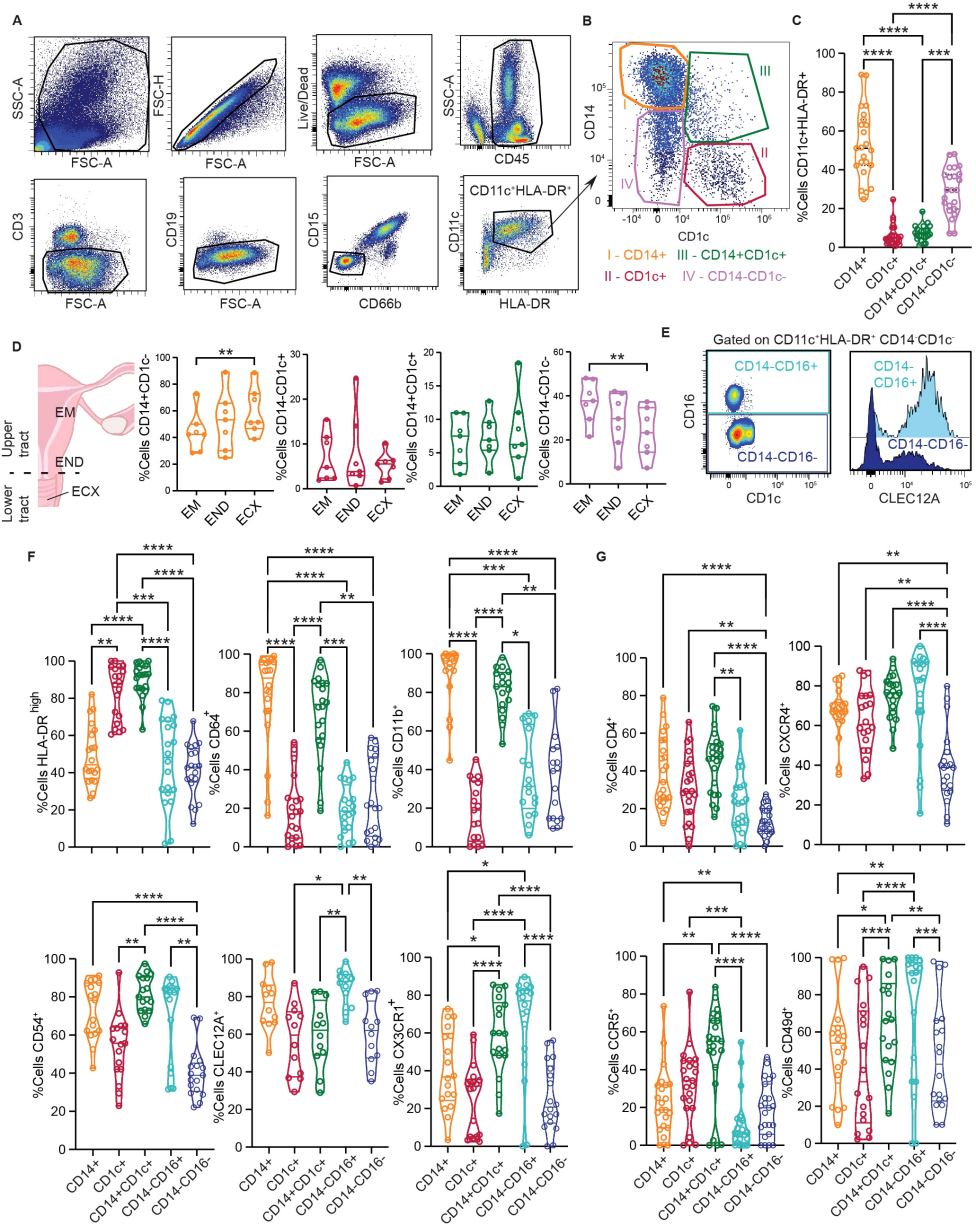
CD14 DCs represent a heterogenous, activated population within the genital mucosa. **(A)** Representative flow cytometry gating strategy to identify FGT resident CD11c^+^HLA-DR^+^ cells. **(B)** Representative plot of HLA-DR^+^CD11c^+^ subsets based on CD14 and CD1c expression; CD14^+^CD1c^-^ (I, orange), CD14^-^CD1c^+^ (II, red), CD14^+^CD1c^+^ (III, green) and CD14^-^CD1c^-^ (IV, pink). **(C)** Comparison of CD11c^+^HLA-DR^+^ subset frequencies in the FGT. **(D)** Comparison of CD11c^+^HLA-DR^+^ subset distribution across different anatomical regions of the FGT, endometrium (EM), endocervix (END) and ectocervix (ECX). **(E)** Representative gating to differentiate CD14^-^CD1c^-^ cells based on CD16 expression (left); Histogram comparing CLEC12A expression between CD14^-^CD16^+^ and CD14^-^CD16^-^ cells (right). **(F)** Comparison of surface protein expression between different CD11c^+^HLA-DR^+^ cells; HLA-DR^high^ (top left), CD64 (top middle), CD11b (top right), CD54 (bottom left), CLEC12A (bottom middle) and CX3CR1 (bottom left). **(G)** Comparison of HIV tropic surface proteins between different CD11c^+^HLA-DR^+^ cells; CD4 (top left), CXCR4 (top right), CCR5 (bottom right) and CD49d (bottom right). Statistics – Friedman’s multiple comparison, (p ≤ 0.05 *; p ≤ 0.01 **; p ≤ 0.001 ***; p ≤ 0.0001 ****).

Data in this study highlights the inherent heterogeneity of DC subsets within the mucosa, with unique transcriptional and phenotypic properties under homeostatic conditions. To validate our CITE-seq findings in a larger number of patients and further delineate genital DC populations, we developed a multi-parameter spectral flow cytometry panel (**Supplementary Table S2**). We gated on CD11c^+^HLA-DR^+^ cells (**Figure 3A**) and identified four different populations based on CD1c and CD14 surface expression (**Figure 3B**), main discriminatory markers identified in our CITEseq data (**Figure 1C**). As seen in **Figure 3C**, CD14^high^CD1c^-^ cells (CD14^+^ DCs) were the most abundant subset (53%), followed by CD14^low^CD1c- (29.1%), CD14^+^CD1c^+^ DCs (DC3s) (7.44%) and CD14^-^CD1c^+^ (cDC2s) cells (6.5%). To determine if CD11c^+^HLA-DR^+^ subset distribution varies throughout the genital tract, we compared the abundance of each subset in the endometrium (EM), endocervix (END), and ectocervix (ECX) (**Figure 3D**). We observed an increased abundance of CD14^+^ cells in the ECX compared to the EM, whereas the opposite was true for the CD14^low^CD1c- subset, with no significant changes observed in CD1c^+^ and CD14^+^CD1c^+^ cells.

In our CITE-seq data set we identified two distinct CD14^-^CD1c^-^ populations with differential expression of CD16 and CLEC12A (**Figure 1C**). Therefore, we further characterized the CD14^-^CD1c^-^ population to determine if this group was composed of two different cell subsets. Consistent with our CITE-seq findings, the CD14^low^CD1c^-^ population contained two different subsets: CD14^-^CD16^+^ and CD14^-^CD16^-^ (**Figure 3E**). Furthermore, these two populations could be distinguished by CLEC12A expression (**Figure 3E**, histogram), suggesting CD14^-^CD16^+^CLEC12A^+^ cells are similar to NCMs, whereas lack of CLEC12A expression in CD14^-^CD16^-^ cells corresponds to the inflammatory/activated monocyte population (infMon).

Next, we analyzed expression of surface markers associated with DC activation status and function (**Figure 3F**). First, we assessed maturation status by gating on HLA-DR^high^ cells consistent with our previous publication (25). We observed that a large proportion of CD14^+^CD1c^+^ and CD1c^+^ cells were HLA-DR^high^, significantly higher than other CD11c^+^HLA-DR^+^ subsets. Compared to the other subsets, CD14^+^CD1c^+^ and CD14^+^ cells showed higher expression of CD11b and CD64, associated with cell adhesion and antigen uptake. CD14^+^CD1c^+^ cells expressed the highest levels of CD54, a molecule involved in enhanced immune synapse formation between DCs and T cells (94). CD14^-^CD16^+^ cells displayed the highest levels of CLEC12A (a myeloid inhibitory receptor), and along with CD14^+^CD1c^+^ cells, showed high expression of CX3CR1, the receptor for CX3CL1 (fractalkine).

Previous studies from our group and others have shown that CD14-expressing FGT DCs preferentially capture HIV viral-like particles (15, 18, 20, 25), indicating subset-specific interactions with HIV. Based on this, we evaluated expression of receptors and coreceptors for HIV in the different subsets (**Figure 3G**). We observed that all three DC subsets expressed significantly higher levels of CD4 and CXCR4 compared to CD14^-^CD16^-^ monocytes. Importantly, CD14^+^CD1c^+^ DCs expressed the highest levels of CCR5, which is crucial for HIV acquisition in the mucosa (95). In addition to the classical co-receptors, CD49d (α4β7 integrin) expression was significantly higher in CD14^+^CD1c^+^ DCs and CD14^-^CD16^+^ monocytes compared to other subsets. CD49d acts as a tissue homing marker and non-classical HIV co-receptor important for mucosal infection (96).

Altogether, our flow cytometry data demonstrates the presence of phenotypically distinct genital DC populations and further supports the presence of CD14+ DCs and DC3s within the CD14+ population.We demonstrate that genital DC3s expressed levels of activation markers similar to conventional cDC2s (CD1c^+^ DCs) and share expression of classical monocyte-derived DC markers with CD14^+^ DCs. Additionally, we demonstrate elevated expression of HIV coreceptors in DC3s compared to other DC populations, suggesting predisposed differential response to HIV within the genital mucosa.

### 3.4 Genital DCs undergo rapid transcriptional changes in response to HIV stimulation

Earlier studies investigated the role of DCs in HIV pathogenesis using monocyte-derived DCs over a time course from 6-48 hours to assess their responses to HIV during viral uptake and infection (22). However, most studies evaluating the role of genital DCs in HIV pathogenesis have investigated events that occur 12 hours or more after viral exposure, focusing on DC susceptibility to HIV infection and *trans-infection* to CD4^+^ T cells (38) (15, 18, 20). We have previously shown that genital DCs release chemokines and antimicrobials in a rapid manner, within 3 hours after HIV stimulation (25), indicating a role for DCs in triggering the initial mucosal innate response against the virus. However, the overall antiviral response induced by HIV immediately following challenge of genital DCs, prior to viral replication, integration, or productive infection, remains uncharacterized.

**Figure 4:**
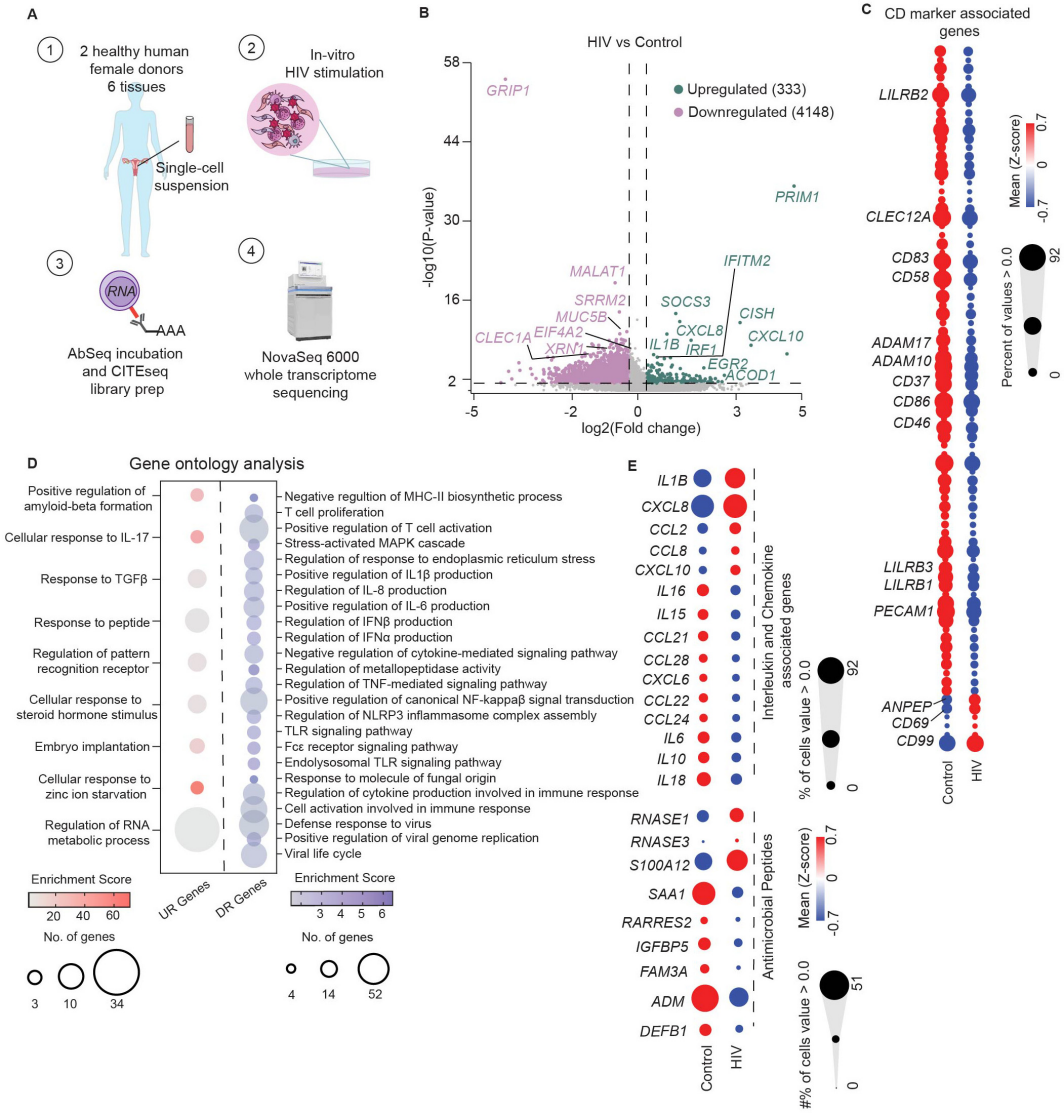
Genital DCs undergo rapid transcriptional changes in response to HIV stimulation. **(A)** Graphical depiction of CITEseq protocol to identify transcriptional changes induced by HIV in FGT resident CD11c^+^HLA-DR^+^ cells. Mixed single-cell suspensions from hysterectomy samples were incubated with HIV at MOI of 0.5 for 30 minutes at 37°C **(B)** Volcano plot of significantly upregulated (n=333) and downregulated genes (n=4148) by HIV in CD11c^+^HLA-DR^+^ cells. **(C)** Hierarchical clustering heatmap comparing gene expression of CD markers between control and HIV exposed cells. **(D)** Bubble-plot visualization of significant GO terms enriched in genes upregulated (UR genes; red) and downregulated (DR genes; blue) in response to HIV by CD11c^+^HLA-DR^+^ cells. **(E)** Hierarchical clustering heatmap comparing genes expression of secreted factors such as interleukins, chemokines and antimicrobial proteins between control and HIV exposed CD11c^+^HLA-DR^+^ cells. Statistics – Volcano plot, non-parametric ANOVA (p ≤ 0.05; -1.2 <FC>1.2); GO terms significance of FDR ≤ 0.05.

To address this gap, we adapted our CITEseq approach to identify early transcriptional responses of genital DCs to HIV. Single cell suspensions generated from human hysterectomy samples were incubated with HIV at an MOI of 0.5 *in vitro* for 30 minutes, prior to proceeding with the CITE-seq protocol described in detail in methods, to determine whole transcriptome profile and surface protein expression simultaneously at the single-cell level (**Figure 4A**). The time of 30 minutes was chosen to identify pathways involved in viral recognition and immune response independent of viral replication, integration and productive infection. We used the surface protein expression information to select CD11c^+^HLA-DR^+^ cells as done in **Figure 1A**, which contains the DC and monocytic populations, and performed differential gene expression analysis between HIV and unstimulated control conditions to define transcriptional changes induced by viral exposure. This analysis identified a significant transcriptional shift induced by HIV within 30 minutes, with 333 genes differentially upregulated and 4148 genes downregulated in response to HIV (**Figure 4B**; (**Supplementary Data Sheet S1**).

To define this HIV response, we first focused on genes associated with DC-mediated immune protection, inflammation, and antiviral response within the top 50 differentially expressed genes based on fold change and p-value (**Figure 4B**; **Supplementary Data Sheet S1**).

Within the upregulated genes, we detected increased expression of the interferon-stimulated genes (ISGs) *IRF1* and *IFITM2*, indicating initiation of innate immune activation and anti-viral response. We also observed a strong upregulation of genes associated with interleukin and chemokine signaling pathways (*IL1B*, *CXCL8*, *CXCL10*, *CISH*, *SOCS3)*, indicating rapid induction of innate secreted responses. Consistent with increased secretory response, we observed upregulation of serglycin gene expression (*SRGN)* in response to HIV, involved in storage and secretion of innate molecules in intracellular vesicles in blood monocytes and other cell types (97). Additionally, we detected upregulation of *ACOD1*, a gene involved in antimicrobial and antiviral responses of innate cells, and a negative regulator of TLR-mediated inflammatory responses (98).

We detected downregulation of genes associated with pro-viral replication of HIV such as *GRIP1*, *EIF4A2, SRRM2, XRN1*, and *MALAT1* (99–102), suggesting suppression of host mechanisms to prevent HIV replication in genital DCs.

To uncover markers that enable the identification of phenotypic changes induced by HIV in genital DCs, we evaluated differences in expression of genes associated with surface protein expression (CD marker list, **Supplementary Data Sheet S2**), focusing on molecules associated with DC function such as activation, migration, and T cell co-stimulation (**Figure 4C**). We observed that the majority of genes related to CD markers were downregulated, but detected strong upregulation of three genes: *CD69*, which mediates tissue retention*; CD99*, involved in diapedesis of monocytes and dendritic cells into inflamed tissue (103); and *ANPEP* (encoding CD13), which mediates DC cross-presentation in human DC populations (104). Analysis of the downregulated genes revealed suppression of markers associated with classical DC activation and T cell co-stimulatory ligands (*CD83*, *CD86, CD58*) and molecules important for migration of DCs (*PECAM1*, *CD37*) (105, 106), indicating potential suppression of T cell interaction molecules and retention of genital DCs within the mucosa at early timepoints. We also observed decreased expression of disintegrin and metalloproteinase family of genes (*ADAM10*, *ADAM17)* which mediate shedding of TNF-α (107), suggesting suppression of classical pro-inflammatory responses. Additionally, relative to the control, the HIV-stimulated DCs had decreased expression of *CLEC12A*, and genes encoding the inhibitory leukocyte immunoglobulin-like receptor subfamily B (*LILRB1*, *LILRB2*, *LILRB3*), suggesting a non-classical mechanism of DC activation (108).

Next, we performed Gene Ontology (GO) analysis of genes upregulated and downregulated by HIV stimulation (FDR ≤ 0.05) (**Figure 4D**; **Supplementary Data Sheet S1**). Analysis of the upregulated genes revealed an enrichment of terms associated with cytokine mediated signaling pathways, innate sensing, and metabolic processes (**Figure 4D**; **Supplementary Data Sheet S1**). Downregulated genes (**Figure 4D**) were associated to terms related to antigen presentation, T cell responses, regulation of classical inflammatory cytokines, type I IFN responses, and canonical proinflammatory pathways, but also suppression of viral cycle and viral replication. These GO terms suggest suppression of classical inflammation but activation of innate host antiviral responses within 30 minutes of exposure to HIV.

Based on these GO terms, we further explored the secretory response to HIV and compared expression of genes encoding cytokines, chemokines and antimicrobials between control and HIV conditions (**Figure 4E**). In response to HIV we detected upregulation of an innate secretory response, including pro-inflammatory genes *IL1B* and *CXCL10*, *CXCL8* and *CCL2*, chemoattractant for neutrophils, DCs and monocytes; and *CCL8*, a CCR5 ligand with antiviral activity, (109). Compared to the control group, we observed decreased expression of genes encoding inflammatory cytokines *IL6* and *IL18*, which act as Th1 polarizing cytokines; *IL16*, a CD4^+^ cell specific chemoattractant; *CCL28*, a CCR10^+^ T cell chemoattractant; and *CCL21*, which mediates homing to lymphoid tissues, suggesting potential inhibition of CCR7+ DC migration towards lymph nodes. Comparison of antimicrobial peptide gene expression ( (110); **Supplementary Data Sheet S2**) revealed increased expression of *RNASE1* and *S100A12* in the HIV group compared to control (**Figure 4E**). Interestingly, we observed suppression of *SAA1* (encoding serum amyloid A), which contributes to induction of pathogenic Th17 cells (111), known to be preferential targets for HIV infection (6, 112, 113). Additionally, we observed suppression of *ADM* (encoding the anti-bacterial peptide adrenomedullin), which also acts as suppressor of inflammatory cytokines (114).

Taken together, our data indicates that mononuclear phagocytic populations respond to HIV in a rapid manner within 30 minutes of exposure, with upregulation of endogenous host restriction factors, activation of non-classical inflammatory responses, and increased gene expression of cytokines, chemokines and antimicrobials, suggesting initiation of a localized antiviral response.

### 3.5 CD14^+^ DCs largely mediate the rapid innate secretory signature observed in genital DC response to HIV while cDC2s mediate antiviral inflammatory responses

**Figure 5:**
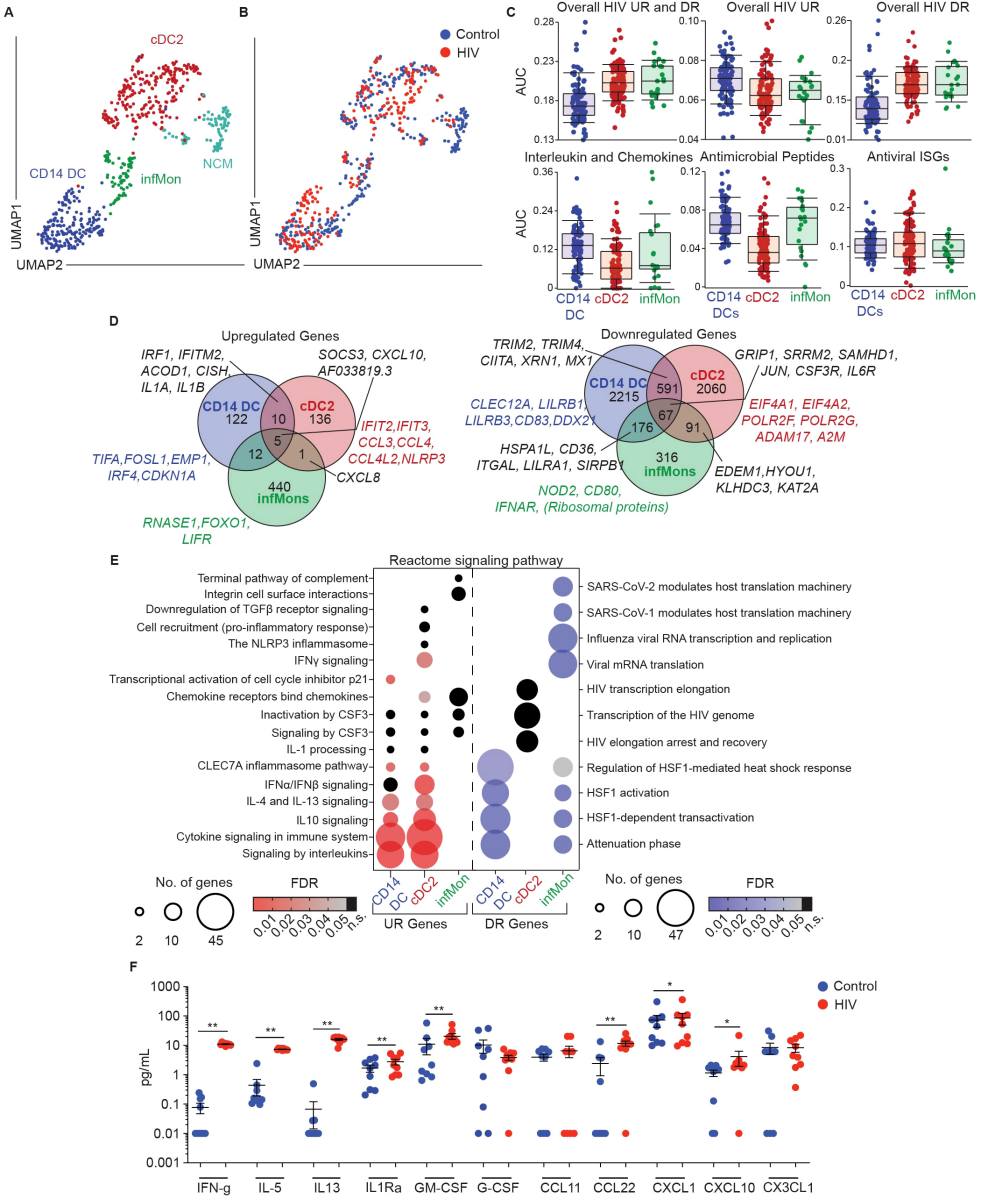
CD14+ DCs largely mediate rapid protective host response observed in genital DC response to HIV. **(A)** Representative UMAP visualization of CD11c^+^HLA-DR^+^ clusters and **(B)** overlay of control and HIV exposed cells (MOI=0.5 for 30 min). **(C)** AUCell comparing enrichment in CD11c^+^HLA-DR^+^ subsets in HIV exposed samples of overall upregulated (UR) and downregulated (DR) HIV signatures (top row) and key gene ontology (GO) terms associated with dendritic cell function (bottom row). **(D)** Venn-diagram representation comparing expression of shared and unique genes significantly upregulated (top) and downregulated (bottom) between CD11c^+^HLA-DR^+^ subsets in response to HIV stimulation. (p-value ≤ 0.05; -1.2 <FC>1.2) **(E)** Bubble-plot representation of biological pathways significantly Reactome significantly enriched in upregulated (UR; red) and downregulated (DR; blue) genes in response to HIV stimulation between different CD11c^+^HLA-DR^+^ subsets. (FDR ≤ 0.05; black dots indicate non-significant (n.s.) FDR values) **(F)** Comparison of secreted protein levels in supernatants of media (control; blue) and HIV exposed (HIV; red; MOI 0.5 for 3h), FGT resident CD14^+^ cells. Significance – Paired non-parametric Wilcoxon-test (p ≤ 0.05 *; p ≤ 0.01 **).

Transcriptional changes in genital mononuclear phagocytic populations exposed to HIV indicates rapid activation of endogenous host restriction factors and localized non-classical inflammatory response, but also an overall suppression of gene transcription. Since our results identified distinct DC subsets with differential homeostatic activation and functions, we next determined how each of these subsets contributes to the overall response after HIV challenge. To define cell clusters and determine their responses following HIV exposure, we used the CITEseq libraries generated from single-cell suspensions stimulated with HIV for 30 minutes shown in **Figure 4**. We performed PCA, followed by unbiased clustering and visualization using UMAP. This analysis revealed four clusters (**Figure 5A**): CD14 DCs, cDC2s, infMons and NCMs, consistent with our findings under homeostatic conditions (**Figure 1B**). Overlay of unstimulated and HIV exposed cells, revealed contribution from control and HIV stimulation conditions to each cluster, except for the NCM cluster, where we found a very limited number of cells from the HIV stimulated condition, and were therefore unable to analyze NCM response to HIV (**Figure 5B**).

First, we utilized the overall transcriptional signature detected after HIV exposure shown in **Figure 4B** to perform AUCell analysis and determine whether this signature was enriched in specific genital DC subsets (**Figure 5C**). Interestingly, the overall HIV response signature (including upregulated and downregulated genes) was preferentially enriched in cDC2s and infMons. However, we observed enrichment of HIV upregulated genes in CD14 DCs, whereas HIV downregulated genes were enriched in cDC2s and infMons, suggesting that HIV challenge preferentially suppresses transcriptional changes in cDC2s and infMons compared to CD14 DCs. Furthermore, we detected an enrichment of cytokine, chemokine and antimicrobial peptide signatures within the CD14^+^ DC population, but no differences in enrichment of antiviral ISGs (**Figure 5C**), suggesting that CD14^+^ DCs largely mediate innate secretory response to HIV at early stages.

We next analyzed differential gene expression within each subset to identify transcriptional changes induced by HIV exposure (**Figure 5D**; **Supplementary Data Sheet S1**). All three subsets shared upregulation of *AF033819.3* (the viral transcript from viral input), *SOCS3*, and *CXCL10* (**Figure 5D**). CD14 DCs and cDC2s shared expression of anti-viral genes *IRF1*, *IFITM2* and *ACOD1* and the cytokine signaling genes *CISH*, *IL1A* and *IL1B*. CD14 DCs and infMons shared expression of zinc finger proteins and non-protein coding genes, except for *CSF3* (encoding the granulocyte colony stimulating factor (G-CSF)) (**Supplementary Data Sheet S1**). cDC2s and infMons both upregulated *CXCL8* expression upon HIV stimulation.

Next, we analyzed genes uniquely upregulated by each subset in response to HIV. We observed that CD14 DCs upregulated *TIFA* and *FOSL1*, genes associated with initiation of inflammation; *EMP1*, previously described in type I interferon stimulated DCs (115); and *CDKN1A*, a gene involved in p53 transcription previously shown to be protective at early stages of HIV infection (116). cDC2s uniquely upregulated ISGs *IFIT2* and *IFIT3*, both possessing antiviral functions, *CCL3*, *CCL4* and *CCL4L2*, encoding chemokines capable of binding HIV receptors (133, 134) and *NLRP3*, encoding inflammasome protein and indicating rapid induction of inflammation. infMons upregulated *RNASE1 (*encoding RNA degrading secreted protein) (117), and *LIFR*, which encodes CD118 described to function as an alternative receptor for HIV (118).

Downregulated genes were more abundant, and we focused our analysis on genes associated with DC function and HIV pathogenesis (**Figure 5D**; **Supplementary Data Sheet S1**). All 3 subsets shared lowered expression of genes associated with HIV transcription such as *GRIP1* and *SRRM2*, which promote HIV replication, and interestingly *SAMHD1*, which plays a role in preventing replication of HIV. Additionally, all three subsets showed decreased expression of inflammation associated genes *JUN, CSF3R* and *IL6R*. CD14 DCs and infMons shared downregulation of *HSPA1L* (encoding a heat shock protein) and genes associated with adaptive immunity such as *CD36*, *ITGAL*, *LILRA1* and *SIRPB1*. CD14 and cDC2s shared suppression of HIV-replication promoting gene *XRN1*, and interferon signaling associated genes *TRIM2, TRIM4, CIITA* and *MX1*. infMons and cDC2s shared downregulated expression of genes associated with unfolded protein response, such as *EDEM1, HYOU1, KLHDC3* and *KAT2A* (119). CD14 DCs uniquely downregulated inhibitory genes *CLEC12A, LILRB1*, and classical DC activation marker *CD83*, suggesting non-canonical activation of this subset. We also observed downregulation of HIV binding and nuclear localization protein encoding gene *DDX21* (120), suggesting inhibition of nuclear import. cDC2s downregulated genes associated with promoting HIV replication such as *EIF4A1*, *EIF4A2* (HIV translation initiation) (121) and RNA polymerase II encoding genes *POLR2F, POLR2G* (122). We also observed downregulation of the TNFα degrading gene *ADAM17* and *A2M* encoding alpha2-macroglobulin, that binds to inflammatory interleukins with high affinity, suggesting inhibition of HIV replication and promoting inflammatory environment. infMons downregulated expression of *NOD2*, *CD80*, and *IFNAR* indicating decreased cell activation.

We performed pathway analysis using Reactome to gain information about biological interactions (50) (**Figure 5E**; **Supplementary Data Sheet S1**). Analysis of upregulated genes revealed shared involvement of CD14 DCs and cDC2s in cytokine signaling pathways (IL-10, IL-4/IL-13 pathways). cDC2s uniquely upregulated Type I IFN and IFN-γ signaling, while CD14 DCs uniquely upregulated transcriptional activation of p21, a known restriction factor against HIV infection in monocyte-derived DCs (123). Analysis of downregulated genes revealed shared signatures between CD14^+^ DCs and infMons for heat shock response, and a unique signature for infMons related to downregulation of viral replication.

Finally, to validate our findings of a secreted response at the protein level, we purified genital CD14 DCs by magnetic bead selection and incubated them with HIV as described in methods. Supernatants harvested 3 hours post stimulation from unstimulated and HIV exposed cells were assayed to quantify secreted proteins (**Figure 5F**). In response to HIV, we detected increased secretion of cytokines and chemokines (IFN-γ, IL-5, IL-13, IL1Rα, GM-CSF, CXCL1, CXCL10 and CCL22). These results confirm rapid innate secretory response in response to HIV.

Overall, we demonstrate subset-specific responses to HIV, with a preferential suppression of genes in cDC2s and infMons compared to CD14 DCs, with CD14^+^ DCs largely mediating the secretory innate response after HIV stimulation.

## 4 Discussion

In this study we evaluated dendritic cell heterogeneity in the human female genital mucosa and how DC subsets respond to HIV immediately after exposure. We found that the CD11c^+^HLA-DR^+^ myeloid population in the genital mucosa includes three DC subsets and two monocyte/macrophage populations with distinct functional and phenotypic properties under homeostatic conditions. However, following HIV exposure, the antiviral response is dominated by DCs’ rapid secretory response, activation of non-classical inflammatory pathways and host restriction factors. Further, we report subset specific differences in genital DC response to HIV, where CD14^+^ DCs are the major subset activated by HIV and responsible for the secretory antimicrobial response, while cDC2s activate inflammasome pathways and antiviral IFN responses. Recognizing that DCs play a crucial role in responding to invading pathogens and recruiting adaptive immunity, identifying DC subsets with anti-HIV properties could aid targeted HIV prevention and vaccination strategies.

We and others have reported the presence of different DC subsets in the genital mucosa (15, 17, 20, 25, 30, 31, 38), however in-depth characterization of these subsets, specifically the CD14-expressing DC compartment, throughout the female genital tract according to recent advances in the field remains lacking (37). Mucosal DC characterization in humans is technically challenging due to their rare nature and the difficulty in isolating specific subsets for functional characterization. To overcome these barriers, we utilized multi-omics approaches to characterize protein surface expression and transcriptome profile of DCs at the single-cell level in mixed cell suspensions from human hysterectomies. Our analysis revealed that the CD11c^+^HLA-DR^+^ population is highly heterogeneous, including multiple DCs and monocyte/macrophage subsets. Within the DCs populations, we identified CD14^+^ DCs, CD1c^+^ cDC2s and CD14^+^CD1c^+^ DC3s, with CD14^+^ DCs representing the dominant population, consistent with prior reports using flow cytometry (25). Importantly, we identified two monocyte/macrophage subsets within CD11c^+^HLA-DR^+^ cells, indicating that CD11c and HLA-DR expression are not sufficient to define DCs in the genital tract.

Under homeostatic conditions the different myeloid populations displayed differential expression of PRRs, chemokines/cytokines and antimicrobials, indicating subset-dependent roles in tissue homeostasis and differential predisposition to sense and respond to pathogens, including HIV. Specifically, PRRs previously associated with HIV membrane binding and detection (such as *CLEC4A*, *CLEC4E*, *TLR2* and *TLR4)* were preferentially expressed by CD14^+^ DCs, suggesting that CD14^+^ DCs may play a principal role in early detection of HIV upon exposure within the genital tract. Although prior studies using *in vitro* generated monocyte-derived DCs demonstrated that DCIR (encoded by CLEC4A) was responsible for viral capture and transinfection to CD4+ T cells (60, 61), future functional studies are needed to test whether DCIR is involved in HIV capture by genital CD14+ DCs. Interestingly, our data indicates a lack of gene expression by CD14+ DCs of other classical HIV-binding lectins (CD207, CD209 and MRC1) which were uniquely expressed in the infMons subset that shares characteristics of macrophages. Further research is needed to define the mechanisms responsible for viral capture by genital DCs. Cytoplasmic and endosomal sensors for viral RNA (*TLR7/8, DDX58, IFIH1*) were enriched in the monocyte/macrophage subsets, possible indicating delayed responses or roles in later HIV detection following viral replication. Cytokine/chemokine expression patterns further pointed for CD14^+^ DCs to play a role in antibacterial defense and inflammatory responses, while cDC2s were involved in maintenance of tissue homeostasis, regulation of inflammation, and promotion of a Th2/T regulatory environment. Overall, this suggests that the CD14^+^ DC population is pre-armed to generate rapid innate responses against incoming pathogens. Interestingly, between the subsets, no differences were observed in antigen processing and presentation, and T cell proliferation pathways, indicating that these populations share their antigen presenting cell properties under homeostatic conditions, consistent with our prior observations (30, 31).

Our validation of this myeloid subset classification using flow cytometry further allowed phenotypical comparisons between the CD14^+^ DC, CD14^+^CD1c^+^ DCs and CD1c^+^ cDC2 populations and the establishment of markers to discriminate the activated monocyte/macrophage populations (CD16, CLEC12A). Consistent with studies evaluating blood cells (35, 36, 59), we found that the CD14^+^CD1c^+^ subset displayed an intermediate phenotype between the monocyte-derived CD14^+^ DCs and the classical myeloid DCs (CD1c^+^ cDC2s), with high expression of HLA-DR but also monocyte origin-associated markers (CD64, CD11b, CX3CR1). This suggests that CD14^+^CD1c^+^ DCs in the genital mucosa are a homolog of DC3s described in peripheral blood and tumors (35, 36, 59), but in the genital tract they are present under steady-state conditions. In addition, the DC3 subset showed increased expression of classical and non-classical HIV coreceptors (CCR5, CD49d, CX3CR1) relevant for mucosal HIV pathogenesis (95, 96, 124), suggesting enhanced ability for viral uptake by CD14^+^CD1c^+^ DC population as described previously by us and others (20, 25). These phenotypic differences in expression of activation, origin, antigen uptake and HIV tropic markers highlight the importance of understanding genital DC subsets to develop targeted strategies.

A novel aspect of our study is the identification of early transcriptional signatures in genital DCs immediately following HIV exposure. While DC infection and trans-infection to T cells have been evaluated previously by others in blood (125, 126) and mucosal tissues (15, 18, 20), the early antiviral responses induced immediately following mucosal HIV exposure remained uncharacterized. In this study, we uncovered that, at early time points, before viral replication takes place, HIV exposure induces a rapid secretory response at the transcriptional and protein levels, activation of host restriction factors (*IRF1, IFITM2, ACOD1*), upregulation of genes involved tissue retention (CD69), and suppression of genes involved in T cell activation (*CD83, CD86, CD58*). Taken together, our data suggests that shortly after exposure, DCs remain in the mucosa and play a role in initiating local innate antiviral protection. However, several inflammatory markers were also upregulated and therefore the consequences for tissue protection and potential attraction and activation of HIV target cells remains to be determined.

Another novel contribution of our study is the discrimination of responses following HIV stimulation in different subsets of genital DCs. We found that all subsets shared activation of genes related to secretion of cytokines and chemokines, although CD14^+^ DCs and cDC2s were the predominant subsets involved in this response. Here, we observed shared upregulation of genes associated with inflammation and antiviral properties (*IRF1, IFITM2, ACOD1, CISH, IL1A, IL1B*) between cDC2s and CD14^+^ DCs upon exposure to HIV. These findings are consistent with earlier studies using monocyte-derived DCs and macrophages which demonstrated the upregulation of IRF1 transcripts and protein, in addition to other ISGs (22, 127). In addition, we uncovered unique pathways elicited by HIV stimulation in each subset. CD14^+^ DCs were the main players in overall antimicrobial defense, responses to TLR activation and initiation of inflammation (*TIFA, FOSL1, EMP1*), while cDC2s displayed a more specific antiviral response with activation of type I interferon (*IFIT2, IFIT3, IRF1*) and inflammasome (*NLRP3*) pathways. Importantly, both subsets activated mechanisms to prevent HIV replication. CD14^+^ DCs induced transcriptional activation of p21, a host restriction factor in monocyte-derived DCs (123), while cDC2s downregulated genes necessary for HIV transcription (*EIF4A1, EIF4A2, POLR2F, POLR2G*). In contrast to the DC subsets, infMons were not involved in initial antiviral protection, but downregulated pathways related to viral cycle, suggesting inhibition of HIV replication. However, future time-course studies are needed to better understand the kinetics and mechanisms by which HIV modifies DC function in a subset-specific manner to promote infection, *trans-infection* and HIV dissemination. Additionally, our study only used HIV-BaL, a laboratory adapted strain, but our findings were not confirmed with HIV transmitted/founder (TF) strains. Although prior studies with DCs and macrophages found no differences between HIV-BaL and TF strains (20, 24, 128), future studies are needed to define DC subset-specific responses to TF strains immediately following exposure to HIV.

Finally, we validated the transcriptional signatures by characterizing the anti-HIV secretory response at the protein level in supernatants from CD14 DCs purified from the genital tract. Despite our observation of upregulation of *IL1B* at the transcriptional levels, we did not detect IL1-β production in our cultures. However, we detected production of IFN-γ, GM-CSF and chemokines with inflammatory and antiviral properties (CXCL10, CCL22). These results complement our prior identification of early secretion of CCR5-ligands and antimicrobial proteins by CD14^+^ DCs in response to HIV (25). Production of GM-CSF, IFN-γ, and CXCL10 has been shown to be induced by TLR stimulation in DCs (129, 130), suggesting that TLR signaling in CD14^+^ DCs may be responsible for induction of cytokine and chemokine secretion. Interestingly, while IFNγ and CXCL10 are involved in mediating Th1 and CD8^+^ T cell adaptive immunity (131, 132), we also detected production of the Th2 cytokines IL5 and IL13, involved in allergic inflammation and activation of Th2 CD4^+^ T cells (135). While we have previously described that under homeostatic conditions CD14^+^ DCs induce proliferation of CD8^+^ T cells with tissue-resident memory phenotype (30), and proliferation of CD4^+^ T cells and double negative (DN) T cells (31), future studies are needed to determine how these cytokine profiles modify tissue environment, susceptibility to HIV infection, and modify T cell induction profile.

Our study has several limitations mainly due to the rare nature of mucosal DCs and the technical difficulties in isolating human DC subsets. First, due to the very low frequency of cDC2s within the genital mucosa, we were unable to isolate cDC2s from mixed cell suspensions to determine their secretory response to HIV at the protein level. Similarly, lack of distinct surface markers and low cell numbers in the genital tract prevented isolating DC3s (CD14^+^CD1c^+^) and resulted in clustering and isolation of this subset together with the CD14^+^ DCs. Therefore, quantifying cDC2 and DC3s individual response to HIV at the protein level will require innovative strategies. Furthermore, our analysis of subset-specific responses was unable to evaluate non-classical monocytes due to low numbers of cells in the HIV stimulated condition, and therefore the contribution of this subset to HIV pathogenesis remains to be elucidated. Despite these limitations, our study provides valuable novel information using an experimental model to evaluate initial mucosal responses to HIV exposure that allows the study of DC subsets without lengthy processing time that could potentially modify primary DCs.

Overall, we demonstrate that the female genital mucosa is populated with different subsets of DCs that specialize under homeostatic conditions and that, immediately following HIV exposure, initiate a local secretory antiviral response and activate host mechanisms to prevent HIV replication in a subset-specific manner. Our findings contribute to the field of mucosal HIV acquisition and provide a map to identify therapeutic targets that trigger local protective innate immune responses against HIV without inducing detrimental tissue inflammation.

## Data Availability

The data presented in the study are deposited in the NCBI GEO repository, accession numbers GSE279408, GSE279774 and GSE279775.
