# Supplementary material for: Human genital dendritic cell heterogeneity confers differential rapid response to HIV-1 exposure
Source: Front Immunol. 2024 Oct 25;15:1472656. doi: 10.3389/fimmu.2024.1472656 (PMC11543421; doi:10.3389/fimmu.2024.1472656)
Supplement: Supplementary file 3 [file Table1.docx]

| **Antibody Tag** | **Catalog #** | **Clone** | **Oligo-nucleotide sequence** |
| --- | --- | --- | --- |
| CD103 | 940067 | BER-ACT8 | AAATAGTATCGAGCGTAGTTAAGTTGCGTAGCCGTT |
| CD274 | 940035 | MIH1 | ATCGTAAGGCTCGTGGTTCGTAAGTAAGTTCGTATC |
| CD69 | 940019 | FN50 | CAATAACGGGTCATAGTAAGTCGCGAGTAAGAGGGC |
| CD152 | 940034 | BNI3 | TAGTATCCGTAGTAGTTATCTGCCCGTTCGTTATGC |
| CD14 | 940005 | MPHIP9 | TGGCCCGTGGTAGCGCAATGTGAGATCGTAATAAGT |
| CD62L | 940041 | DREG | ATGGTAAATATGGGCGAATGCGGGTTGTGCTAAAGT |
| CD10 | 940045 | HI10A | CCTGTTTGATGCGTACGGAGATTTAGCGGATTTATG |
| CD16 | 940006 | 3G8 | TAAATCTAATCGCGGTAACATAACGGTGGGTAAGGT |
| CD184 | 940056 | 12G5 | CAGTGTTTAGAGCGGGTTGCATATGTCGTTTAGAGG |
| CD163 | 940058 | GHI/61 | TATTATGTGCGAACTATGGTATCCGTATTGAGGGCT |
| CD1a | 940063 | HI149 | TTGGTTGCAGTGCGGTCGAAGATACGTAGTGAGATT |
| CD195 | 940050 | 2D7/CCR5 | ATGGTTTAGTCGTACGTGGGTTTAGATTGGCGGTGC |
| CD83 | 940054 | HB15e | AAGCTTGGACGATGGTATATTAACGATTGAGAGTGC |
| CD1c | 940083 | F10/21A3 | ATAGATTACATTCGTTTAGCGTTGGGTTCGGTCCGT |
| CX3CR1 | 940216 | 2A9-1 | GGGTTCACGAGGTTTAAAGCGGTAGTATAGGATGCC |
| HLA-DR | 940235 | TU39 | GAATCGAGTTTATAGGTGGCGTTAGTAGTTGTGGGC |
| CD19 | 940247 | HIB19 | AAGCGGTAAATCGGGAGTAAGTCGTGTTCTAGCAGT |
| CD117 | 940250 | 104D2 | ATGGAGAGCGATTGCGTGAGGATATGCGAGATTGTT |
| CD11c | 940265 | S-HCL-3 | GTCGGTTCGTGATTTAGTTAGTGCGTCTTAGTGTCC |
| CD11b | 940266 | ICRF44 | ATGGATTCGGTTCGGTGTTTGGATAGATAGGCTGCG |
| CD279 | 940467 | MIH4 | ACGAGAAATGCGCGGAATGGGTGAGTTAGTAAGACG |
| BDCA-2 | 940282 | V24-785 | TAAGGTAGGCAGTAGATAACGGGACGAATGATGAGC |
| CD4 | 940304 | RPA-T4 | AATGTGCGGCGGTGTATATGATCGAGTCCAACGTCT |
| CD8 | 940305 | SK1 | AGGACATAGAGTAGGACGAGGTAGGCTTAAATTGCT |
| CD3 | 940307 | UCHT1 | AGCTAGGTGTTATCGGCAAGTTGTACGGTGAAGTCG |
| CCR7 | 940394 | 2-L1-A | AATGTGTGATCGGCAAAGGGTTCTCGGGTTAATATG |
| CD15 | 940274 | W6D3 | ATAGGCATGGACGACGTAGATAATAAGTGGCGGGTT |
| CD64 | 940262 | MD22 | ATGTAGTCTGTATAGCGGTGTAGCGGATTAAAGGCG |
| CD127 | 940012 | HIL-&R-M21 | AGTTATTAGGCTCGTAGGTATGTTTAGGTTATCGCG |

**Supplementary Table 1.** List of oligo-conjugated surface protein antibodies (AbSeq) used for CITEseq experiments.
