# Supplementary material for: Human genital dendritic cell heterogeneity confers differential rapid response to HIV-1 exposure
Source: Front Immunol. 2024 Oct 25;15:1472656. doi: 10.3389/fimmu.2024.1472656 (PMC11543421; doi:10.3389/fimmu.2024.1472656)
Supplement: Supplementary file 4 [file Table2.docx]

| **Target** | **Clone** | **Fluorophore** | **Catalog Number** | **Vendor** |
| --- | --- | --- | --- | --- |
| CD45 | H130 | BUV395 | 563792 | BD Biosciences |
| Viability | - | Live/Dead Blue | L23105 | Thermo Fisher |
| CD16 | 3GB | BUV496 | 612944 | BD Biosciences |
| CD19 | SJ25C1 | BUV563 | 612916 | BD Biosciences |
| CD64 | 10.1 | BUV737 | 612776 | BD Biosciences |
| CD4 | SK3 | BUV805 | 612887 | BD Biosciences |
| CD54 | HA58 | BV421 | 353132 | BioLegend |
| CD66b | G10F5 | Pacific Blue | 305112 | BioLegend |
| CD14 | MφP9 | BV480 | 566141 | BD Biosciences |
| CD3 | REA613 | VioGreen | 130-113-142 | Miltenyi Biotec |
| CD11b | ICRF44 | BV570 | 301315 | BioLegend |
| CD49d | SK11 | BV711 | 304332 | BioLegend |
| CCR7 | G043H7 | BV750 | 353254 | BioLegend |
| CXCR4 | 12G5 | BV785 | 306530 | BioLegend |
| CD8 | SK11 | SparkBlue | 344760 | BioLegend |
| CD15 | H198 | PerCP-Cy5.5 | 301922 | BioLegend |
| CD1c | L161 | PE | 331506 | BioLegend |
| CX3CR1 | 2A9-1 | PE-eF610 | 61-6099-42 | Thermo Fisher |
| CCR5 | 2D7/CCR5 | PE-Cy5 | 556889 | BD Biosciences |
| CLEC12A | 50C1 | APC | 353606 | BioLegend |
| HLA-DR | LN3 | AF700 | 327014 | BioLegend |
| CD11c | Bu15 | APC-Cy7 | 337218 | BioLegend |

**Supplementary Table 2.** Fluorescence-conjugated antibodies used for spectral flow cytometry experiments.
